# Measurement of tear resistance after manual capsulorhexis and femtosecond laser–assisted capsulotomy of crystalline lenses

**DOI:** 10.1007/s10103-021-03448-1

**Published:** 2021-10-29

**Authors:** Mandy Patzlaff-Günther, Michael Fromm, Thomas Kern, Martin Bartram, Anna Schwäblein, Dag Heinemann, Sonja Johannsmeier, Carsten Framme, Tammo Ripken

**Affiliations:** 1 Industrial and Biomedical Optics Department, Laser Zentrum Hannover E.V, Hannover, Germany; 2 Rowiak GmbH, Hannover, Germany; 3 Department of Ophthalmology, Hannover Medical School, Hannover, Germany; 4 Hannover Centre for Optical Technologies, Leibniz University Hannover, Hannover, Germany; 5 Cluster of Excellence PhoenixD, Leibniz University Hannover, Hannover, Germany; 6 Lower Saxony Centre for Biomedical Engineering, Implant Research and Development, Hannover, Germany; 7 Institute of Horticultural Production Systems, Leibniz University Hannover, Hannover, Germany

**Keywords:** Cataract surgery, Continuous curvilinear capsulorhexis, Femtosecond (fs)-laser-assisted capsulotomy, Lens refilling, Tear resistance, Phacoemulsification

## Abstract

**Background:**

In this study, the tear resistance of porcine lens capsules after continuous curvilinear capsulorhexis (CCC) and femtosecond (fs)-laser-assisted capsulotomy for cataract surgery (FLC) with different laser parameters is measured with a custom-made testing setup.

**Methods:**

Forty-five fresh porcine lenses were randomly chosen for CCC (*n* = 15) or FLC 1 (*n* = 15) and FLC 2 (*n* = 15). The FLC 1-group was treated with smaller spot distances than the FLC 2-group. The force necessary to break the opening of the anterior capsule and the maximum displacement were measured.

**Results:**

The mean tear resistance of the CCC-group (150 ± 70 mN) was higher than that of the FLC 1-group (60 ± 20 mN) and the FLC 2-group (30 ± 20 mN).

**Conclusion:**

It could be shown that CCC leads to a significantly higher tear resistance of the opening than FLC in porcine lenses. The femtosecond laser group demonstrated that smaller spot distances lead to a higher tear resistance.

## Introduction

In 2020, cataract was the leading global cause of blindness in people aged 50 years and older [1]. This eye disease is caused by a clouding of the crystalline lens resulting in blurred vision. Cataract surgery is the most frequently performed operation on the human eye. The WHO estimated the global number of cataract surgeries at 32 million for 2020 [2]. Due to the increasing human life expectancy, the number of cataract-affected eyes is also growing rapidly as this disease occurs mainly with advanced age [3].

Until now, there is no medical treatment available to stop or reverse cataract. The only way to restore the patient’s vision is the operative replacement of the crystalline lens with an artificial lens.

The gold standard involves opening the lens anterior capsule manually with a so-called continuous curvilinear capsulorhexis (CCC). Femtosecond (fs)-laser-assisted cataract surgery (FLC) has been established as an alternative technique since 2010 [4]. After opening the capsule, the internal lens is emulsified via ultrasound or laser lens fragmentation and the opacified lens tissue is then aspirated. Finally, the intraocular lens (IOL) is inserted in the capsular bag.

For an optimal insertion of the IOL into the capsular bag and a reduction of the complication risk after the operation, it is important to ensure a high accuracy concerning centration, size, and circularity of the cut and to guarantee a high tear resistance [5] [6] [7].

One clear advantage of the more expensive and time-consuming FLC in comparison to CCC is the more precise realization of the geometry of the cut, which ensures that the IOL is contained in the capsular bag close to the effective lens position (ELP) [8]. Contradictory data has been published concerning tear resistance. Some studies show a higher tear resistance of CCC in comparison to FLC [9] [10], while others show the opposite result [11] [12]. In this study, a testing device for measuring tear resistance of lens capsules after CCC in comparison to FLC with different laser parameters was realized and the results were evaluated. A higher tear resistance is connected to a lower risk of damage of the capsular bag for the patient. The goal of this study is to form a valid statement concerning the more effective operation method, especially in relation to the laser parameters.

## Material and methods

### CCC and FLC on porcine samples

Fresh porcine eyes from approx. 6-month-old pigs were obtained from two different local abattoirs few hours after slaughter. Porcine globes were kept in preserving medium at room temperature (22 °C) and the experiments were run within 12 h of the death of the pigs. The eyes were randomly selected for CCC and FLC. The cornea and iris were removed. Afterwards, CCC or FLC was conducted in air with direct access to the crystalline lens.

To perform the manual capsulorhexis, the capsule was punctured with a curved needle. The opening was treated with a thin forceps (similar to utrata forceps) so that the tissue was torn in a circle. The diameter, measured after the procedure, was about 4 ± 1 mm. The opening diameter of the capsulotomy with the laser has been adapted to that of the manual capsulorhexis, since a constant opening can be generated with the laser more easily than during manual capsulorhexis.

The capsulotomy was conducted with a fs-laser (FCPA μJEWEL D-400, IMRA America Inc., Ann Arbor, USA) with a wavelength of 1040 nm, a pulse duration of 306 fs, and a spot size of approximately 5 µm. The diameter of the capsulotomy was 4 mm. The exact position of the anterior capsule was detected via an integrated optical coherence tomography (OCT) system. The pulse energy was 2 µJ.

**Fig. 1:**
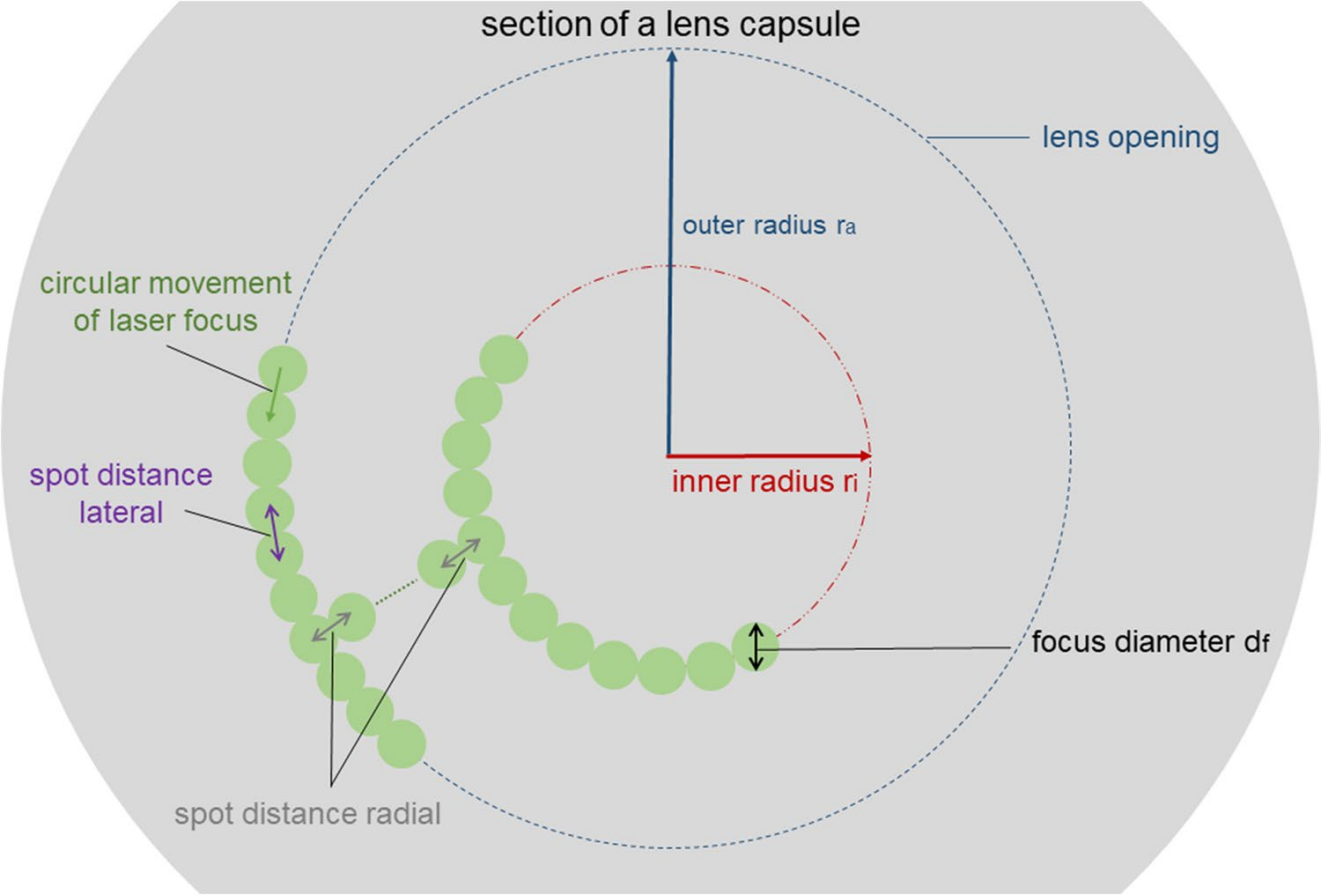
Simplified geometry (only one plane of the z-stack) of fs-laser capsulotomy pattern (parameter set B) with the most important parameters. See also Table 1

The applied capsulotomy pattern consisted of concentric cylinders with an inner diameter of 1.8 mm and an outer diameter of 2.0 mm. The “spot distance radial” indicates the distance between two adjacent cylinders. The “spot distance lateral” is the distance between two adjacent spots on the cylinder ring and the “spot distance depth” indicates the spot distance in the axial direction. In the FLC-group, two different sets of spot distances were used (see Table 1). A pulse overlap in radial, lateral, and axial direction is generated in parameter set A; the spot distances are increased in parameter set B (see Fig. 1).

**Table 1:** Laser parameter for capsulotomy (FLC 1, FLC 2)

	Parameter set A (FLC 1)	Parameter set B (FLC 2)
Outer radius *ro* (mm)	2.0	2.0
Inner radius *ri* (mm)	1.8	1.8
Spot size *df* (μm)	5	5
Spot distance lateral (μm)	3	5
Spot distance radial (μm)	3	5
Spot distance depth (μm)	10	15
Pulse energy (μJ)	2	2
Pulse length (fs)	306	306

For each group (CCC, FLC 1, FLC 2), *n* = 15 eyes were used. After opening the anterior capsule, the inner lens was emulsified with a standard phaco-machine (Millennium, Bausch&Lomb).

### Phacoemulsification

After opening the anterior lens capsule, the internal lens was emulsified with a standard phaco-machine (Millennium, Bausch Lomb). For this purpose, the lens nucleus was destroyed with a vibrating needle through the lens opening via ultrasound (US). Afterwards, the lens fragments were aspirated with a pump. The parameters used for phacoemulsification are shown in Table 2.

**Table 2:** Parameters used for phacoemulsification

Parameter	Value
Position of infusion holder	55 cm
Max. vacuum	100 mmHg
Max. US	15%

**Fig. 2:**
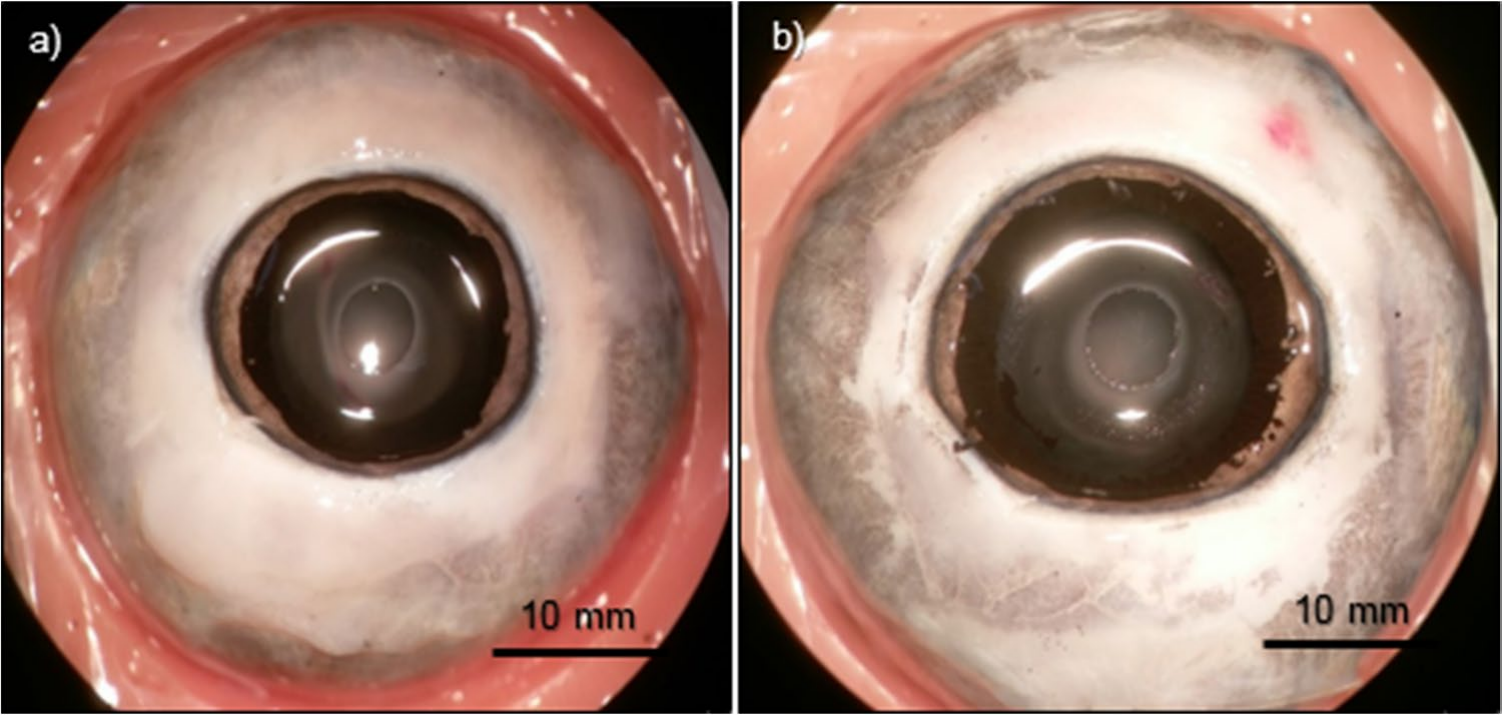
Porcine eye globes after phacoemulsification with CCC (**a**, diameter 4 ± 1 mm) and FLC with parameter set A (**b**, diameter 4 mm)

Figure 2 shows two porcine eye globes after phacoemulsification with CCC (a) and FLC (b).

### CCC on human sample

To compare the results of the porcine lenses to those of human donors, we had the chance to perform tear resistance with the same setup following a CCC on one human donor lens (74 years old). The lens was obtained as part of an ongoing study (Ethics vote No. 7303—Untersuchung des Akkommodationsverhaltens von ex-vivo Humanlinsen nach fs-Laser-Behandlung und sog. “Lens-Refilling”). As the human capsule is thinner than the porcine capsule, the CCC was more difficult to perform and turned out less circular. Moreover, the lens was already separated from the globe so that phacoemulsification was not possible. Therefore, the mechanical measurement was performed on the filled lens.

### Mechanical test

**Fig. 3:**
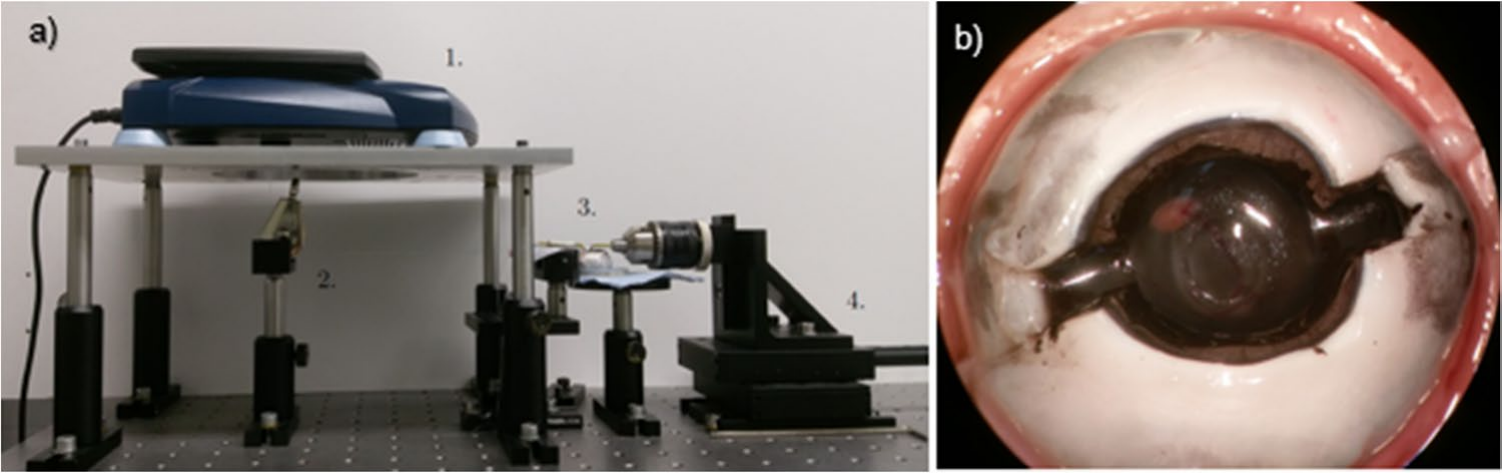
**a** Custom-made testing setup with scale (1), nylon thread for the connection of the left hook to the scale (2), left and right hook (3) and linear stage (4), **b** sclera incisions to minimize the resistance of the sclera to the stretching

Mechanical testing was performed with a custom-made testing setup shown in Fig. 3a. The setup consisted of a scale (Scout-Pro, OHAUS, Nänikon, Switzerland) for tensile force measurement with a range from 0 to 2000 g and a resolution of 0.1 g (equal to 0.981 mN) and a linear stage (Z825B, Thorlabs, Inc., NJ, USA) with a resolution of 29 nm.

On each side (stage and scale), a metal hook (1.55 mm diameter) was mounted. For the testing, two incisions in the sclera were made at the positions of the metal hooks. This was done to minimize the resistance of the sclera to the stretching (see Fig. 3b). For the measurement of tear resistance, the linear stage was moving one of the hooks with a constant velocity of 0.2 mm/s. The eye globe was bedded in oil, and was able to move with the movement of the linear stage transferred to the globe. In the reference system of the globe, both hooks were moving with equal velocity and were effectively stretching the capsule opening until it teared. Displacement and stretching force were recorded during the whole stretching process via video. The displacement was recorded in millimeters with a precision of four decimals and the weight load in grams with a precision of one decimal. The resulting strain *ε* in percent was calculated via the following formula:

1 $$\varepsilon =\frac{{U}_{1}}{{U}_{2}}*100\%$$

where ${U}_{1}$ is the circumference of the opening with the radius $r$ before stretching:

2 $${U}_{1}=2*\pi *r$$

and ${U}_{2}$ the circumference of the opening at the point of tear after a stretching distance $l$ calculated by:

3 $${U}_{2}={U}_{1}+2*l$$

### Analysis

To calculate the statistical significance of the results, an unpaired *t* test was performed [13]. In an unpaired *t* test, two independent samples are compared [14]. In this work, the results of tear resistance of CCC and FLC were compared. Then the p-value was calculated [15]. A *p* value smaller than 0.05 was interpreted as a statistically significant result.

## Results and discussion

### CCC and FLC on porcine samples

**Fig. 4:**
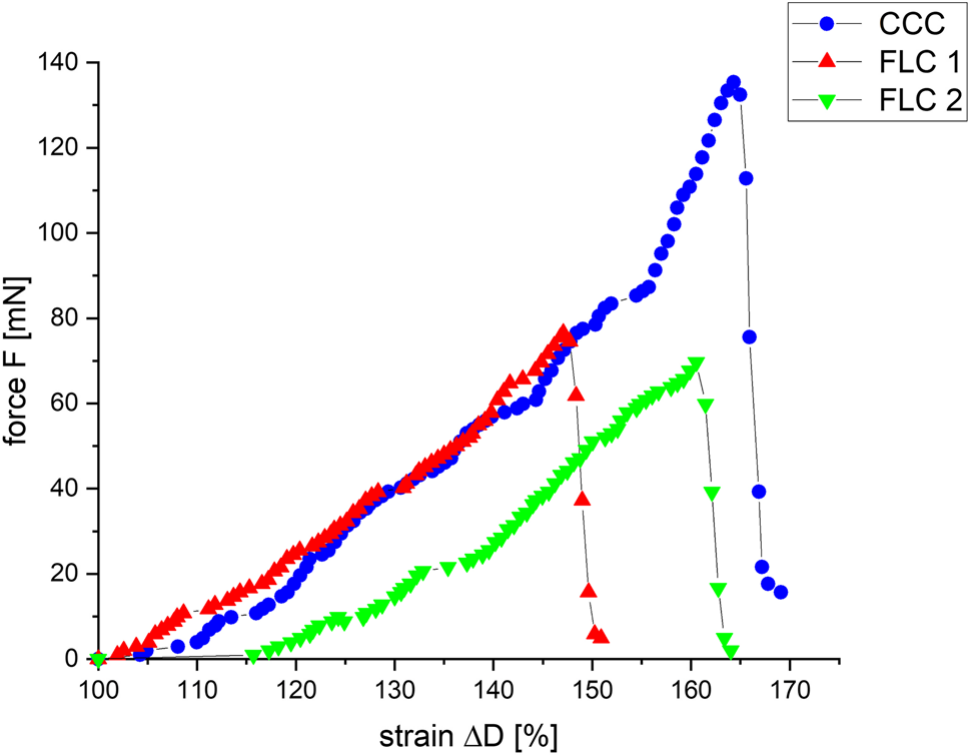
Example plots of tear resistance measurements. Traction force as a function of strain for porcine lenses. Blue: after CCC (max. force = 135 mN); red: after FLC 1 (max force = 77 mN); green: after FLC 2 (max force = 70 mN)

The traction force as a function of strain is shown for one example of each of the three series of experiments (CCC, FLC 1, FLC 2) in Fig. 4. All data show an exponential increase of the force with stretching up to a certain point where the lens capsule tears.

The results of the three series of measurements of tear resistance are shown in Table 3. The mean tear resistance of the CCC-group (series 1, 150 mN ± 70 mN) was higher than that of the FLC 1-group (series 2, 60 mN ± 20 mN), and the FLC 2-group (series 3, 30 mN ± 20 mN). The FLC 1-group showed a higher tear resistance than the FLC 2-group.

**Table 3:** Results of tear resistance measurements with calculated mean values of the three experimental series for porcine lenses (*n* = 15)

Number of eye	CCC (mN)	FLC 1 (mN)	FLC 2 (mN)
1	108	85	29
2	107	61	25
3	132	116	31
4	156	82	46
5	174	52	20
6	188	58	31
7	187	44	37
8	73	52	21
9	311	39	34
10	129	55	9
11	236	32	10
12	217	13	10
13	79	46	36
14	47	48	70
15	135	77	15
Mean value	150	60	30
Standard deviation	70	20	20

The greater variability in maximum force to capsule tear in the CCC group could be due to the form of the CCC, which is approximately but not perfectly circular in comparison to the FLC group. However, there was no correlation of the measured values of maximum force to the capsulotomy size.

**Fig. 5:**
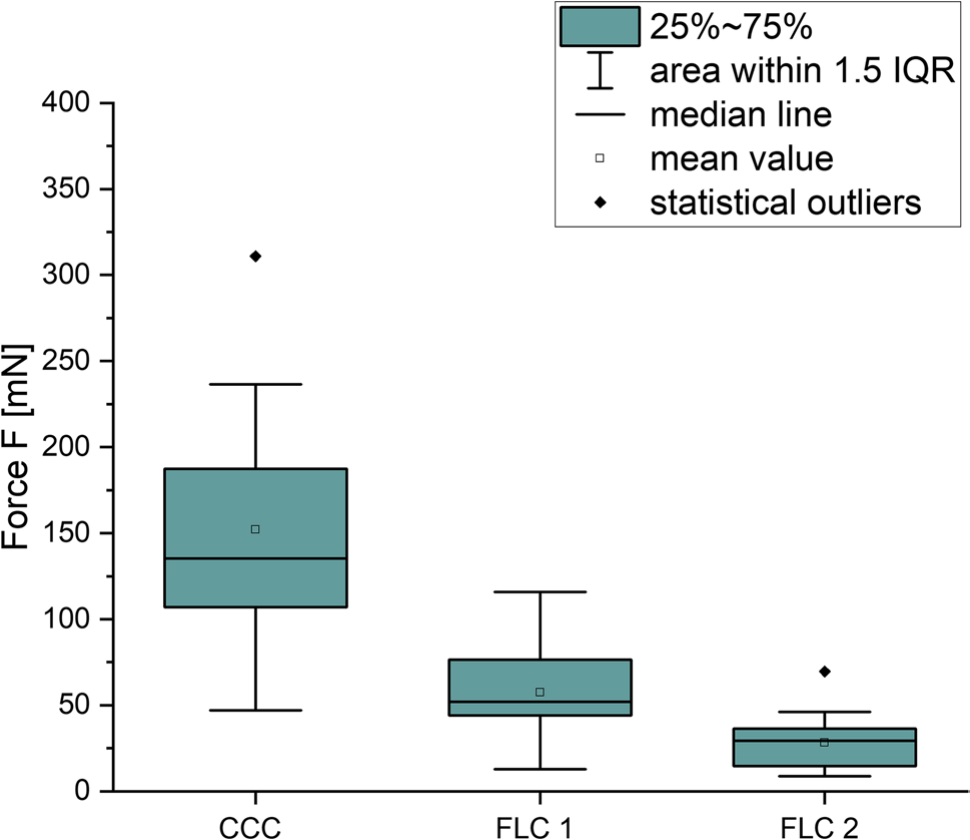
Box plot of maximum force before tear for the three measurement series (CCC, FLC 1, FLC 2)

In Fig. 5, a box plot with the maximum force before tear of the opening for each lens capsule is shown.

A *t* test analysis of the values of the CCC- and the FLC 1-groups showed a statistically significant difference (*p* < 0.0001). Furthermore, the difference between the FLC 1- and the FLC 2-group was statistically significant (*p* = 0.0007) but smaller than the difference between the CCC- and the FLC 1-group. It can also be seen that the standard deviations are higher in the CCC-group (± 70 mN) than in the FLC-groups (± 20 mN).

The higher tear resistance of the CCC-group compared to the FLC-groups confirms the results of Sándor et al. [10]. The value of the maximum force before tear for the manual capsulorhexis (150 mN ± 70 mN) is in good agreement with the results of Sándor et al. [10] as well (155 mN). The force value for the capsulotomy (FLC 1) measured in this study (60 mN ± 20 mN) was found to be lower than that of Sándor et al. [10] (119 mN) who used a LenSx femtosecond laser (Alcon Laboratories, Inc. Forth Worth, TX) with different parameters (pulse energy: 5 µJ, spot distance radial and lateral: 4 µm, spot distance depth: 3 µm). However, these results are hardly comparable without knowing all laser parameters (e.g. spot size, pulse overlap).

In contrast, the results of Auffarth et al. [11] and Friedman et al. [12] showed a lower tear resistance of the porcine lens capsule after manual capsulorhexis (65–73 mN) than after capsulotomy (113–152 mN). The tear resistance of the lens capsule after laser capsulotomy was higher in both studies (113–152 mN) than the result of this work (60 mN ± 20 mN). Auffarth et al. measured 113 mN with a pulse energy of 5 μJ, spot size of approximately 5 to 10 μm, a spot separation of 5 μm, and a line separation of 2 μm [11]. The study of Friedman et al. showed an increasing tear resistance with decreasing pulse energy from 113 mN (10 μJ) to 152 mN (3 μJ) [11].

One assumption which would explain why FLC could lead to a lower tear resistance than CCC is the uneven edge of the cut after capsulotomy. This unevenness is due to the perforations of the single laser pulses in the tissue compared to the smooth edge after CCC. This was also demonstrated in Scanning Electron Microscope (SEM) examinations of the lens capsule edge in the work of Sándor et al. [10]. The lens capsule tears faster because of the predetermined breaking points produced with the laser. The smaller the distances between the laser perforations and the smaller the applied energies, and thus the breaking points, are, the smoother is the edge of the opening. This is confirmed by the present work, where the FLC 1-group showed higher tear resistances than the FLC 2-group. In a clinical study of Schultz et al. [16] on 100 eyes, a horizontal spot distance of 5 µm was used which falls within the range of our parameters. It was shown that with a vertical spot distance of 15 µm, the capsulotomy quality was improved in comparison to 10 µm. However, as there is no information on the spot size, this result must be interpreted with caution. In a study of Daya et al. [17], it was shown that continuous circular manual capsulorhexes are more resistant to tear than discontinuous manual capsulorhexes as well.

One reason for the differences of the absolute results of tear resistance in our work compared to other studies might be the operator-dependent quality and shape of the CCC on the one hand and the spot distances and spot overlap in combination with the pulse energy used in the FLC on the other hand.

Differences in the preparation of the pig eyes for the test series e.g. in storage could also have an impact on the laboratory study regarding differences in rupture force and stretching ratios.

In this study, CCC and FLC were conducted in air. The cornea and iris were removed before permitting free access to the anterior lens capsule. In the clinical practice, contact lenses or fluid interfaces on the cornea are used; thus, the focal size might be a little bit larger and the absolute laser energy needed to cut a capsulotomy would be slightly higher. This could result in a slightly different tensile strength.

The laser–assisted and manual capsulotomy in the study of Auffarth et al. was performed through the cornea with the complete eye globe intact. For the tear resistance measurements, the cornea was then removed and the anterior capsule was cut out. In the work of Friedman et al., the capsular bag was filled with a low-viscosity liquid containing 0.05% gelatin after phacoemulsification, which also has an effect on the stability of the capsule. In the present study, however, the capsule remained in the eye during the measurements. This could lead to greater stability of the capsular bag.

A significant difference between the porcine model and human lenses is the much thicker lens capsule in pigs (approx. 60 µm) [18], compared to the human lens capsule (approx. 11–15 µm depending on age) [19]. This has a direct influence on the absolute tear resistance values, and the porcine capsule is more difficult to tear than the human capsule.

Finally, with regard to the procedure of the capsulorhexis in the present study, it has to be noted that the entire cornea and partial iris are missing. In clinical surgery, the stability of the anterior chamber is exploited. The chamber is evenly filled with a viscoelastic agent before starting the rhexis. This exerts pressure from the anterior towards the posterior of the anterior chamber during the rhexis, which ideally prevents the rhexis from running out. This could also have an impact on the absolute values of tear resistance.

All in all, these results suggest that it should be possible with optimized laser parameters (low pulse energy and small spot distance) to further increase the tear resistance of capsulotomies done with the fs-laser. This will be the scope of future work in this field.

### CCC on human sample

The maximum force for the human sample performed with CCC was 20 mN at a strain of 128%. It shows an exponential increase of the force with stretching up to a certain point where the lens capsule tears like the porcine lenses, but with a considerably lower maximum force. As mentioned before, the human capsule is thinner than the porcine capsule. That might be the main reason for the lower maximum force. The measured tear resistance agrees well the published value of 25.8 mN for a human lens [20]. In the future, more experiments on human lenses should be conducted with FLC while altering the laser parameters, and the results are to be compared with CCC.

## Conclusion

In this work, the tear resistance of porcine lens capsules after different opening methods was measured with a custom-made testing setup. It was shown that the highest tear resistance is reached by opening the capsule with a manual capsulorhexis (mean 150 mN ± 70 mN). Opening the capsule with a femtosecond laser with the given parameters resulted in a significantly lower tear resistance. One possible reason for this is the uneven edge of the cut after capsulotomy, arising from the perforations of the single laser pulses in the tissue compared to the smooth edge after CCC. Inside the femtosecond laser group, the tear resistance of FLC 2 (larger spot distances) was even lower (mean 30 mN ± 20 mN) that of the FLC 1 (pulse overlap, mean 60 mN ± 20 mN). This work clearly shows the correlation between the tear resistance of the opening of the anterior capsule after FLC and the pulse overlap. The higher the distances between the laser-induced predetermined breaking points are the earlier the tissue tears. Moreover, it should be possible to produce smoother edges with FLC using lower pulse energy and more pulse overlap.

Furthermore, the tear resistance, centration, circularity, and size of the opening in the anterior capsule play an important role concerning the final position of an IOL [5] [6] [7]. During a manual capsulorhexis, this is strongly dependent on the expertise and performance of the surgeon. In this respect, the fs-laser shows a clear advantage and delivers reproducible results, as can be seen in the higher standard deviations related to CCC in comparison to FLC in this study.

The measurement of the tear resistance of one human donor lens was in good agreement with previously published measurements. In the future, more measurements of tear resistance should be conducted to compare CCC and FLC in human lenses with different sets of laser parameters (spot distance, spot size, pulse energy) and different cutting patterns. Thereby, the dependence of the surface roughness of the cut and the tear resistance of the capsule opening can be analyzed more in detail.

## Data Availability

All data are fully available without restriction. The data underlying the results presented in the study are available from m.patzlaff-guenther@lzh.de.
